# Predictive medicine: outcomes, challenges and opportunities in the Synergy-COPD project

**DOI:** 10.1186/1479-5876-12-S2-S12

**Published:** 2014-11-28

**Authors:** Felip Miralles, David Gomez-Cabrero, Magí Lluch-Ariet, Jesper Tegnér, Marta Cascante, Josep Roca, Synergy-COPD consortium

**Affiliations:** 1 Barcelona Digital Technology Centre, Barcelona, Spain; 2 Unit of Computational Medicine, Department of Medicine, Center for Molecular Medicine, Karolinska Institutet, Karolinska University Hospital, Stockholm, Sweden; 3 IDIBAPS, Hospital Clínic. Departament de Medicina. Facultat de Medicina and Departament de Bioquimica i Biologia Molecular, Facultat de Biologia. Universitat de Barcelona. Barcelona, Spain; 4 Departament de Bioquimica i Biologia Molecular i IBUB, Facultat de Biologia, Universitat de Barcelona, 08028 Barcelona, Spain; 5 CIBERES. Centro de Investigación en Red de Enfermedades Respiratorias (CibeRes), Palma de Mallorca; 6 Departament d'Enginyeria Telemàtica (ENTEL), Universitat Politècnica de Catalunya, C/Jordi Girona, 1-3, C3, 08034 Barcelona, Catalonia, Spain

**Keywords:** chronic diseases, co-morbidities, chronic obstructive pulmonary disease, information and communication technologies, modelling, network medicine, training

## Abstract

**Background:**

Chronic Obstructive Pulmonary Disease (COPD) is a major challenge for healthcare. Heterogeneities in clinical manifestations and in disease progression are relevant traits in COPD with impact on patient management and prognosis. It is hypothesized that COPD heterogeneity results from the interplay of mechanisms governing three conceptually different phenomena: 1) pulmonary disease, 2) systemic effects of COPD and 3) co-morbidity clustering.

**Objectives:**

To assess the potential of systems medicine to better understand non-pulmonary determinants of COPD heterogeneity. To transfer acquired knowledge to healthcare enhancing subject-specific health risk assessment and stratification to improve management of chronic patients.

**Method:**

Underlying mechanisms of skeletal muscle dysfunction and of co-morbidity clustering in COPD patients were explored with strategies combining deterministic modelling and network medicine analyses using the Biobridge dataset. An independent data driven analysis of co-morbidity clustering examining associated genes and pathways was done (ICD9-CM data from Medicare, 13 million people). A targeted network analysis using the two studies: skeletal muscle dysfunction and co-morbidity clustering explored shared pathways between them.

**Results:**

(1) Evidence of abnormal regulation of pivotal skeletal muscle biological pathways and increased risk for co-morbidity clustering was observed in COPD; (2) shared abnormal pathway regulation between skeletal muscle dysfunction and co-morbidity clustering; and, (3) technological achievements of the projects were: (i) COPD Knowledge Base; (ii) novel modelling approaches; (iii) Simulation Environment; and, (iv) three layers of Clinical Decision Support Systems.

**Conclusions:**

The project demonstrated the high potential of a systems medicine approach to address COPD heterogeneity. Limiting factors for the project development were identified. They were relevant to shape strategies fostering 4P Medicine for chronic patients. The concept of Digital Health Framework and the proposed roadmap for its deployment constituted relevant project outcomes.

## Introduction

Synergy-COPD (2011-14) [1] was an European Union project, within a call dedicated to the Virtual Physiological Human 7^th ^Framework Program, conceived to explore the potential of systems medicine to generate knowledge on underlying mechanisms of chronic obstructive pulmonary disease (COPD) heterogeneities observed in the patients both in terms of clinical manifestations and disease progression [2,3]. A core component of the project was to transfer of the acquired knowledge into the clinical arena with a twofold purpose. Firstly, analysis of the role of a systems approach to COPD heterogeneity to enhance individual health risk assessment and stratification leading to innovative patient management strategies. The second purpose was to identify novel modalities for the interplay between healthcare and biomedical research aiming at fostering deployment of 4P Medicine for patients with chronic disorders [4-6]. Ultimately, Synergy-COPD was designed to generate outcomes in three different dimensions: (i) biomedical area; (ii) information and communication technologies (ICT); and, (iii) transfer into healthcare.

**Biomedical area **- The central biomedical hypothesis in Synergy-COPD was that the classical *"spill over" *theory [7,8] supporting that COPD heterogeneity is driven by the pulmonary events of the disease is not valid. Alternatively, we proposed that COPD heterogeneities result from the interplay of mechanisms governing three conceptually different phenomena: 1) pulmonary disease, 2) systemic effects of COPD and 3) co-morbidity clustering, each of them with their own dynamics. To this end, a network medicine approach [9-11] was used to investigate the underlying mechanisms of two different actionable phenomena showing negative impact on prognosis of COPD patients: (i) skeletal muscle dysfunction/wasting, as a hallmark of systemic manifestations of the disease [12]; and, (ii) co-morbidity clustering in these patients [13-15]. Two subsidiary biomedical aims were: (i) to explore shared abnormalities between the two phenomena, skeletal muscle dysfunction and risk of co-morbidity clustering; and, (ii) to investigate the relationships between skeletal muscle dysfunction and nitroso-redox disequilibrium [12] with focus on assessing the potential of a quantitative analysis of the relationships between cellular hypoxia and abnormal reactive oxygen species (ROS) generation.

**Targeted ICT developments **- Three well differentiated ICT developments were carried out during the project lifetime. Firstly, the COPD Knowledge-Base (COPDkb) from clinical and experimental data, text-mining results and public databases [16]. The COPDkb combines genetic information with molecular, physiological and clinical data. It provides data integration and semantic mapping connected with mathematical modelling and the Simulation Environment [17]. The second group of ICT developments was associated with different types of mathematical modelling [18] aiming at addressing the biomedical questions alluded to above. They encompassed different types of: (i) qualitative and semi-quantitative network analyses; (ii) classical mechanistic modelling based on ordinary differential equations (ODE), as well as (iii) research on the interplay between network analysis and mechanistic modelling. The third ICT product was the Simulation Environment (SE) [17] aiming at supporting biomedical researchers to concurrently run heterogeneous modelling approaches that may have complementary roles in exploring a given biomedical phenomenon.

**Transfer to healthcare **- A last and most important aspect of the project was the transfer of the acquired knowledge, as novel rules for patient management, into the clinical practice through the generation of clinical decision support systems (CDSS) embedded into the clinical processes defined within different integrated care services (ICS) for chronic patient management [19,20]. The project also assessed two different deployment strategies for these CDSS, in Barcelona and in the United Kingdom.

The ambition of Synergy-COPD, and the complexities faced during the project lifetime, generated deviations from the initial working plans and forced subsequent adaptations that often resulted in an added value for the entire operation. It is of note that the triggering factors generating project deviations covered a wide spectrum of characteristics that are systematically addressed below. The current manuscript: (i) summarizes the main outcomes of Synergy-COPD; (ii) analyses the lessons learnt during the project lifetime; (iii) identifies some of the key challenges to be faced for a successful implementation of 4P medicine for chronic patients; and, (iv) characterizes opportunities for further developments in systems medicine.

## Biomedical outcomes

The central biomedical hypothesis has been described above and it was extensively presented in [19,21] wherein COPD heterogeneity is analysed in detail. The biomedical rationale of the entire project was based on the results of an unbiased clustering analysis of approximately 342 clinically stable COPD patients characterized after their first hospitalization and followed-up during a 5-year period [22]. The PAC_COPD study [22] identified and prospectively validated three COPD subtypes: (i) **Group I**, severe respiratory COPD; (ii) **Group II**, moderate respiratory COPD patients in whom the most distinctive trait was a dissociation between severe emphysema score together with mild to moderate airway remodelling leading and moderate airflow limitation, as expressed by forced expiratory volume during the first second (FEV_1_); and, (iii) **Group III**, including COPD patients in whom the most characteristic trait was co-morbidity clustering, mainly cardiovascular disorders (CVD), type II diabetes mellitus (T2DM) often accompanied by metabolic syndrome (MS). It is of note that skeletal muscle dysfunction was a transversal characteristic with patients distributed in all three PAC_COPD groups [22]. The main biomedical findings of the studies carried out in Synergy-COPD were:

**Abnormal regulation of relevant skeletal muscle biological pathways **- Integrative multilevel analyses of skeletal muscle of healthy subjects and COPD patients [23-26] including different "omics" layers (transcriptomics, epigenetics, proteomics and metabolomics), physiological characteristics and clinical information generated strong evidence of abnormal regulation of muscle bioenergetics both at baseline and, in particular, after the perturbation of the biological system by a standard endurance training protocol. Abnormal training-induced adaptations were observed at several different levels of the mitochondrial respiratory chain, but also in the interplay between oxidative and glycolytic pathways, as well in fatty acids metabolism. The network analysis indicated abnormalities in key metabolic pathways governing skeletal muscle bioenergetics [24] and ribosome biogenesis [27] such as mTOR [12,28-30] and its interplay with insulin signalling pathway [31]. Interestingly, the different analyses carried out in COPD patients consistently showed abnormal relationships between cytokines and tissue remodelling at baseline and after training [24,32].

**Increased risk of co-morbid conditions in COPD patients **- Using 13 million health records from U.S. Medicare [33,34], the project identified 27 disease groups (DG) with significantly elevated risks to co-occur with COPD that increased with ageing. These groups included both well-established associations like CVD or lung cancer, but also unexpected ones, like digestive track disorders, that could be interesting candidates for more focused follow-up investigations. For each DG, we constructed a comprehensive list of known associated genes from the literature and by performing a pathway enrichment analysis a number of pathways that are shared between different disease groups were identified, suggesting that the observed co-morbidities are indeed rooted in shared molecular mechanisms. By further inspecting the genes within prevalent pathways, the project was able to identify a number of genes with the potential to characterize COPD co-morbidities. On-going analyses on potential biomarkers predicting the level of co-morbidity remain to be validated in further studies.

Overall, the biomedical results seem to support the central hypothesis of the project indicating that abnormal regulation of pivotal pathways at systemic level can contribute to both co-morbidity clustering and skeletal muscle dysfunction in these patients. Moreover, the analyses support a potential causal role for nitroso-redox disequilibrium contributing [35] to the abnormal pathway regulation as observed in COPD. Accordingly, individual susceptibility to deregulation of metabolic pathways, together with potential epigenetic mechanisms likely associated with aerobic capacity, may play a role modulating both systemic effects and co-morbidity clustering in these patients, beyond well known risk factors such as tobacco smoking. Despite that the results from different animal experiments carried out during the project lifetime support the above interpretations of the current results, we fully acknowledge that further validation of the current speculations is required before we move to transfer of knowledge into the clinical scenario, as discussed in the following sections of the current manuscript.

An ancillary aim of the project was the analysis of group II of the PAC_COPD study [22] using the mechanistic model of pulmonary spatial heterogeneities as described in [19] and in [36] in order to generate rules for identification of this subset of COPD patients in primary care. The rationale behind this approach is that this subset of COPD patients could be a candidate for screening programs for early diagnosis of lung cancer which is one of the priorities in respiratory medicine. The literature appears to indicate that dissociation between high emphysema score and mild airway remodelling is associated with a higher probability of developing lung cancer [37,38]. Unfortunately, the maturity of the modelling development did not allow completion of the analyses as initially planned.

## Targeted ICT developments

**COPD knowledge base (COPDkb) **- During the project lifetime, the COPDkb [16,39] has been extended to integrate over 40 public data sources on functional interaction (e.g. signal transduction, transcriptional regulation, protein-protein interaction, gene-disease association). Also, integration of COPD-specific expression and co-morbidity networks connecting over 6.000 genes/proteins with physiological parameters and disease states has been accomplished. Three mathematical models describing different aspects of systemic effects of COPD were connected to clinical and experimental data. The technical architecture of the user interface was enhanced in order to provide browser-based access and form-based searches, as described in detail in [39]. A network search enables the use of interconnecting information and the generation of disease-specific sub-networks from general knowledge. Integration with the Synergy-COPD Simulation Environment [17] enables multi-scale integrated simulation of individual computational models. The current COPDkb [39] is the only publicly available knowledge resource dedicated to COPD which combines genetic information with molecular, physiological and clinical data, as well as mathematical modelling. Its integrated analysis functions provide overviews about clinical trends and connections while its semantically mapped content enables complex analysis approaches. The COPDkb has been planned to be offered as a repository to publish and semantically integrate data from relevant clinical trials. It is currently freely available after registration at *www.copdknowledgebase.eu*.

**Modelling achievements **- The project combined several modelling approaches, probabilistic [40] and mechanistic [41-43], to address the three biomedical analyses alluded to above, namely: (i) skeletal muscle dysfunction; (ii) co-morbidity clustering, as well as (iii) group II from the PAC_COPD study [22], as indicated in [19]. Moreover, novel modelling techniques, Bayesian analyses [44] and Thomas network formalism [45,46], addressing the interplay between probabilistic and mechanistic modelling were explored aiming at expanding the potential of future systems-oriented analyses of biological phenomena. A detailed description of the project's achievements in this area can be found in [18].

All in all, both the modelling developments and the strategies adopted showed to be useful to explore the biomedical challenges of the project and to identify potential biomarkers. Moreover, the interplay between the two main modelling strategies indicate the potential of probabilistic modelling, Thomas formalism [45,46], to contribute to parameter refinement in mechanistic modelling (e.g. enhanced parameter estimation for mitochondrial function in the integrated model, [18]. Also, predictive modelling showed potential to assess the multi-scale impact of the outcomes generated by mechanistic modelling (e.g. consequences of abnormally high mitochondrial ROS generation on inflammatory responses or tissue remodelling). However, some limitations in modelling developments and in their clinical impact due to different reasons shall be acknowledged. Main lessons learnt in this area, as well as the associated challenges identified during the project lifetime are described in the following section of the current manuscript.

**Simulation environment **- It was developed as a supporting tool for bio-researchers aiming at studying COPD by running concurrent computational models. The Simulation Environment is composed of two main modules to control and coordinate information-related processes. One of these modules is the graphical visualization environment, the interface through which the user interacts with the models. The other module is the simulation workflow management system. Detailed functionalities and interoperability with the COPDkb are described in detail in [39]. Lessons learnt through the assessment of the Simulation Environment are also discussed in the following section.

## Transfer to healthcare

As indicated above and described in detail in [18], the modelling achievements of the project were far from being mature for clinical application, such that subject-specific predictive modelling generating rules to directly feed clinical decision support systems (CDSS) could not be developed, as initially planned. However, the research team agreed that the main objectives of the transfer to healthcare could be achievable by combined consolidated knowledge on COPD with emerging knowledge from the project. The latter, despite the lack of proper validation, could be useful to generate CDSS aiming at filling well identified clinical gaps. It is fully acknowledged that those CDSS should be further evaluated for clinical use beyond the project lifetime. As discussed in the following section, the strategy finally adopted allowed to overcome some of the limitations of the modelling and facilitated the identification of key bottle necks for future implementation of 4P medicine.

The research team identified two main areas to be considered under the current subheading analysing the transfer to healthcare: (i) steps associated to CDSS generation and deployment; and, (ii) factors modulating the interplay between healthcare and systems-oriented biomedical research.

**Generation of CDSS in Synergy-COPD **- The following three main steps should be considered for the development of CDSS: (i) generation of the rules feeding each CDSS either automatically from computer modelling and/or through the rationale provided by existing knowledge; (ii) clinical validation and acceptability of the rules by the clinical users; and (iii) interoperability with the ICT platform supporting integrated care services (ICS) implying that the CDSS are embedded into the clinical process and show both usability and applicability. In the project, three different families of CDSS, encompassing: (i) diagnosis; (ii) stratification; and (iii) management of COPD with a patient-oriented approach, were conceived with different degrees of development and validation.

Firstly, the suite of CDSS included under the umbrella of the COPD case finding program covers four well defined aims: (i) articulates informal care (pharmacy offices) and formal care (primary care) for early diagnosis of COPD, (ii) ensures remote, web-supported, quality control of testing and assistance to non-specialized professionals; (iii) provides accessibility and interoperability of the testing across healthcare tiers; and, (iv) explores the potential for both regional deployment of the forced spirometry testing program, as well as generalization of the approach to other testing procedures to enforce the transfer of complexity to primary care and to the community. To this end, developments done within Synergy-COPD were articulated with achievements from previous deployment projects [47,48] which demonstrated to be highly productive. Briefly, all key aspects of the COPD case finding program had both qualitative and robust quantitative evaluations, as reported in [20]. The regional deployment of the program in Catalonia was initiated by November 2013 and its complete clinical validation through a large cluster RCT is expected to be completed by the end of 2015.

A second block corresponds to the suite of CDSS enhancing applicability of the 2011 GOLD update [19,20], as well as proposals for stratification of COPD patients. These CDSS are addressing three main goals: (1) applicability of GOLD within an integrated electronic health record; (ii) facilitate future comparative analyses among different approaches for assessing health risk in COPD and deciding on subject-specific stratification; and, (iii) address early disease interventions with emphasis on life-style being physical activity, nutrition and enhanced adherence through self-management the target ones. In summary, this suite of CDSS should support the design of early cost-effective interventions with preventive purposes, facilitate the interplay between community (informal care), primary care and with specialized professionals. This family of CDSS was conceptually developed and qualitatively assessed, but it is not technological in place mainly because deployment is modulated by two main on-going processes: (i) build-up consensus among primary care teams; and, (ii) achievement of interoperability at health system levels. Finally, the third block of CDSS corresponds to community-based management of patients with advanced disease stages currently in the pipeline for regional deployment. The three layers of CDDS provide a coherent spectrum of actions enhancing management of chronic patients.

**Interplay between healthcare and systems-oriented biomedical research **- The project identified that deployment of 4P medicine involving implementation of sound strategies for patient health risk assessment and stratification generating two key requirements. Firstly, articulation between informal care and healthcare allowing incorporation of non-clinical information into patient management; that is, data on patient adherence profile, lifestyle (physical activity, nutritional habits, addictions), social support needs, environmental and societal factors that have a well accepted impact on health status and survival. The use of the personal health folder (PHF) enriched with accredited, customized apps that are properly interoperable with the electronic health record (EHR) may cover a twofold aim: (i) articulation of non-clinical and clinical information; and, (ii) empower patients for self-management.

A second major requirement is the articulation of the scenario described above (PHF + EHR) with a novel type of systems-oriented biomedical research platform ensuring continuous cross-fertilization between research and patient care. This ideal setting would cover both incorporation of non-biomedical data into predictive modelling and provide dynamic modelling approaches that should facilitate the way toward truly personalized medicine. Such a proposal has been described in more detail in [6], under the concept of Digital Health Framework (DHF), wherein the roadmap for its stepwise deployment is analysed.

## Main challenges beyond Synergy-COPD

Major lessons learnt from the analysis of the limitations faced during the development of the project can be grouped in three major clusters: (i) available datasets; (ii) maturity of the field; and, (iii) need for societal changes. The assessment of these three areas helped to identify major challenges to be faced beyond Synergy-COPD:

**Available datasets **- Despite the current exponential generation of large amounts of biomedical data of different nature, several factors associated to availability of appropriate datasets have limited the transfer of the project outcomes on mechanisms of skeletal muscle dysfunction and co-morbidity clustering closer to clinical application. The following limiting aspects were identified: (i) the fragmented nature of the available datasets; (ii) insufficient context-specific information; and, (iii) the lack of large datasets with proper experimental designs including multilevel "omics" information and clinically-driven hypotheses.

Additional problems encountered throughout the project lifetime have been: (i) insufficient harmonization of medical coding across countries and within large longitudinal datasets [49-51]; (ii) gaps in semantic interoperability with large variations in disease definitions and coding; (iii) publicly available information biased toward well established and expected diseases and their underlying mechanisms [52-54]; (iv) lack of multilevel "omics" information bridging between GWAS information and phenotypic characterization [55]; and, (v) lack of accompanying non-clinical information (environmental, lifestyle, socioeconomic factors) in bio-banking data. In summary, the characteristics of the available datasets had a negative impact on the project precluding the generation of subject-specific predictive modelling. But, they also constituted a limitation to validate the explored novel modelling approaches (e.g. Bayesian analysis and Thomas formalism) that should facilitate the interplay between probabilistic and mechanistic modelling for further characterization of complex biological processes. In this regard, policies promoting data-sharing are highly recommended.

**Maturity of the field **- Mechanistic modelling techniques have shown usefulness to characterize biological mechanisms and to provide quantitative assessment of the phenomena analysed [18], but they have serious limitations to address complex biological phenomena. In contrast, network medicine approaches [34,40] based on statistical predictive models [56,57] seem suitable to address complex biomedical phenomena when large amount of data are available. Moreover, high-throughput analysis [58,59] have shown that canonical analysis of biological pathways is too simplistic not reflecting the real complexity of interconnectedness of biological networks [60]. It is of note, however, that the high expectations generated by emerging high-throughput methods are not balanced by a sufficient degree of maturity yet [61,62].

**Societal changes **- The project clearly identified that major organizational and technological changes are required to pave the way for a credible transition toward 4P medicine. Some of the key requirements for such a transition are described in [6] within the concept of Digital Health Framework. But, cultural factors such as: (i) workforce preparation, (ii) evolving concepts in terms of ethical factors relative to privacy of information transfer and information sharing; and, (iii) development of novel business environments fulfilling the requirements of the novel scenario are relevant elements to be taken into account in the definition of strategies leading to a successful implementation of the change.

It must be emphasized that the identification of the limiting elements alluded to above does not define at all a negative landscape for systems-oriented research in the biomedical area. On the contrary, one of the most important outcomes of Synergy-COPD has been the identification of the challenges to be faced and the definition of innovative strategies to adequately overcome the limiting factors alluded to above that should lead to unprecedented developments in the medical practice.

## Opportunities identified during the project lifetime

A profound change in the health paradigm is being fostered by the convergence of three major driving forces originated by the needs for: (i) coping with the epidemics of chronic conditions through implementation of the Chronic Care model; (ii) achieving cost-containment of healthcare through generation of health efficiencies; and, (iii) transferring to clinical practice a mechanisms-based approach to disease based on systems medicine [9,63]. All in all, the emerging scenario is exceedingly favourable for the convergence between integrated care and systems medicine as an efficient way to accelerate a mature deployment of 4P medicine for chronic patients. The outcomes of the Synergy-COPD project clearly reinforce such an orientation for future developments in the field.

The acknowledgment of the complexities faced during the project lifespan delineates the need for planning a building-blocks strategy in future endeavours designed to achieve further progress in the area. Moreover, the concept of Digital Health Framework provides the rationale for prioritization of the ICT developments, as identified in the proposed roadmap for the deployment.

It is currently well accepted that the Chronic Care model through deployment of integrated care services supported by information and communication technologies (ICS_ICT) can contribute to enhance health outcomes without increasing overall costs of the health system. Such a generation of healthcare efficiencies is partly achieved by the transfer of complexities from specialized to primary care and to the community. It is reasonable to hypothesize that the generation of health efficiencies can be markedly boosted by: (i) promoting a more active role of citizens, patients and carers in self-management and co-design of the services; and, (ii) fostering cost-effective preventive strategies aiming at modulating disease progress. These two strategic proposals require adoption of the novel health paradigm that involves bridging traditional healthcare delivery (i.e. formal care) and informal care (e.g. patient self-management, wellness programs, social care, etc.) through adoption of citizen's (patient's) personal health records as management tools. In this new scenario, the correct articulation of patient gateways and mobile devices (mHealth) [64] is promising to empower, for the first time, an efficient channel enhancing accessibility to the health system, facilitating monitoring and including patient's behavioural and environmental factors into health management. The ultimate goal of patient gateways is to support cost-effective preventive interventions to modulate the evolution of the disease which might represent tremendous sources of efficiencies if in-place.

Moreover, the analysis of the lessons learnt during the Synergy-COPD project facilitates the identification of specific challenge-driven opportunities in all the areas described above. A proper prioritization of future actions following the general recommendations generated by the project should contribute to make 4P medicine for chronic patients a successful reality.

## Conclusions

The Synergy-COPD project has demonstrated that a systems-oriented research on COPD heterogeneity generated novel knowledge, not achievable through classical methods. In the project, COPD was targeted as a use case because of the high prevalence and impact of the disease, as well as the relevance of COPD heterogeneity for subject-based health risk assessment and stratification in the clinical arena. Moreover, COPD showed an elevated potential for generalization of the research findings to other prevalent chronic disorders.

The concept of Digital Health Framework developed in the project and the roadmap for its implementation involves an overall strategy for the transition from current healthcare practice to a novel scenario fostering cross-talk between informal care, healthcare system and systems-oriented biomedical research that shall facilitate implementation of 4P medicine for chronic patients.

## Competing interests

The authors declare they have no competing interests.

## Declarations

Publication of this article has been funded by the Synergy-COPD European project (FP7-ICT-270086).

This article has been published as part of *Journal of Translational Medicine *Volume 12 Supplement 2, 2014: Systems medicine in chronic diseases: COPD as a use case. The full contents of the supplement are available online at http://www.translational-medicine.com/supplements/12/S2.
